# Curcumol potentiates celecoxib-induced growth inhibition and apoptosis in human non-small cell lung cancer

**DOI:** 10.18632/oncotarget.23308

**Published:** 2017-12-14

**Authors:** Fangfang Cai, Minghui Chen, Daolong Zha, Peng Zhang, Xiangyu Zhang, Nini Cao, Jishuang Wang, Yan He, Xinxin Fan, Wenjing Zhang, Zhongping Fu, Yueyang Lai, Zi-Chun Hua, Hongqin Zhuang

**Affiliations:** ^1^ The State Key Laboratory of Pharmaceutical Biotechnology, School of Life Sciences, Nanjing University, Nanjing, China; ^2^ State Key Laboratory of Quality Research in Chinese Medicines, Macau University of Science and Technology, Macau, China; ^3^ Nanjing Industrial Innovation Center for Pharmaceutical Biotechnology, Nanjing, China

**Keywords:** celecoxib, curcumol, synergism, apoptosis, tumor metastasis

## Abstract

Combinatorial therapies that target multiple signaling pathways may provide improved therapeutic responses over monotherapies. Celecoxib and curcumol are two highly hydrophobic drugs which show bioavailability problems due to their poor aqueous solubility. In the present study, we evaluated the effects of celecoxib and curcumol alone and in combination on cell proliferation, invasion, migration, cell cycle and apoptosis induction in non-small cell lung cancer (NSCLC) cells using *in vitro* and *in vivo* experiments. Our data showed that the sensitivity of a combined therapy using low concentration of celecoxib and curcumol was higher than that of celecoxib or curcumol alone. Suppression of NF-κB transcriptional activity, activation of caspase-9/caspase-3, cell cycle G1 arrest, and inhibition of survival MAPK and PI3K/AKT signaling pathway contributed to the synergistic effects of this combination therapy for induction of apoptosis. Additionally, either celecoxib alone or in combination with curcumol inhibited NSCLC cell migration and invasion by suppressing FAK and matrix metalloproteinase-9 activities. Furthermore, the combined treatment reduced tumor volume and weight in xenograft mouse model, and significantly decreased tumor metastasis nodules in lung tissues by tail vein injection. Our results confirm and provide mechanistic insights into the prominent anti-proliferative activities of celecoxib and/or curcumol on NSCLC cells, which provide a rationale for further detailed preclinical and potentially clinical studies of this combination for the therapy of lung cancer.

## INTRODUCTION

To date, lung cancer still represents the leading cause of cancer-related deaths worldwide and non-small cell lung cancer (NSCLC) comprises about 85% of all types of lung cancer [1], with only a small number of patients achieving long-term survival. For patients with NSCLC, traditional radiotherapy or chemotherapy has reached its plateau in efficacy, and the search for new treatment strategies is urgently needed.

**Figure 1:**
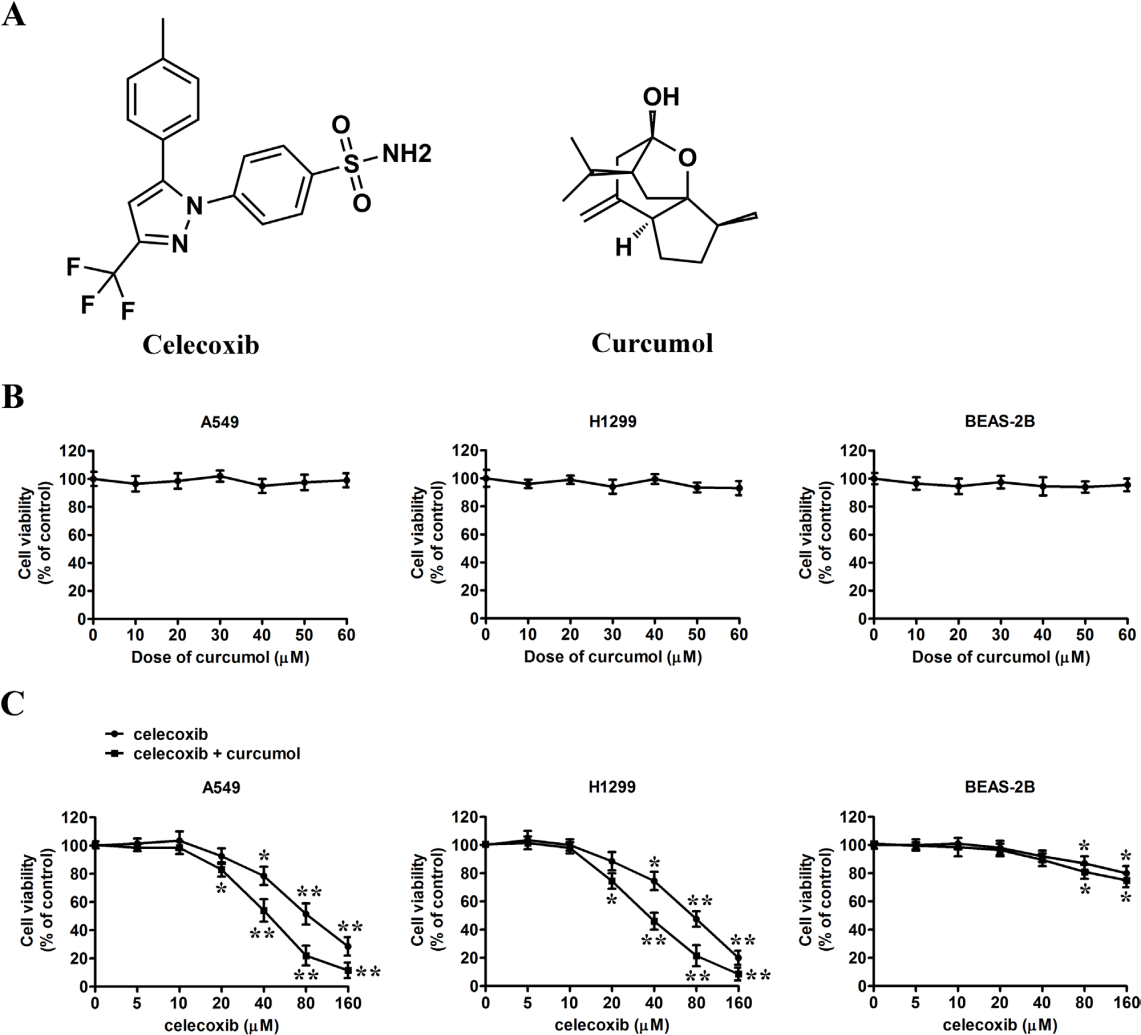
Effect of celecoxib and curcumol on the growth of tumor cells *in vitro* **(A)** Chemical structure of celecoxib and curcumol. **(B)** A549 cells, H1299 cells and BEAS-2B cells were treated with curcumol at different concentrations (0, 10, 20, 30, 40, 50, 60 μM) for 24 h, and the cell viability was assessed by MTT assay. **(C)** A549 cells, H1299 cells and BEAS-2B cells were treated with indicated concentrations (0, 5, 10, 20, 40, 80, 160 μM) of celecoxib for 24 h in the absence or presence of 30 μM curcumol. The cell viability was assessed by MTT assay. Data are represented as mean ± SD. ^*^p < 0.05, ^**^p < 0.01.

Non-steroidal anti-inflammatory drugs (NSAIDs) are a heterogeneous group of drugs associated with inhibition of the inflammation process, mainly targeting enzymes such as cyclooxygenase (COX), responsible for converting arachidonic acid into prostaglandins (PG). COX-2 has been reported to participate in angiogenesis, inflammation, proliferation and tumor growth. Increased COX-2 expression is closely associated with poor angiogenesis and differentiation in many tumor types including lung cancer, and COX-2 activation is related to multidrug resistance and cancer invasion [2–5]. Since COX-2 plays important roles in carcinogenesis and neoplastic progression, NSAIDs are agents of interest for cancer prevention [6]. Celecoxib (for its structure, see Figure 1A), a new NSAID approved by the U.S. Food and Drug Administration that specifically inhibits COX-2 activity without inhibiting COX-1, has been reported to prevent carcinogenesis in both humans and animals [7–9]. The mechanism underlying the effects of celecoxib might be related to COX-2-dependent or -independent pathways [10, 11]. Celecoxib could evoke cell cycle arrest, anti-angiogenesis [12], and cell apoptosis [13, 14] in cancers. However, depending on the rate of administration and the dosage, celecoxib can also have negative side effects [15]. Currently, celecoxib is being widely investigated for therapeutic activity against a wide variety of tumor types as a single agent or in combination with other agents in clinical trials [16, 17].

In recent years, more and more cancer therapeutics in preclinical trails or on the market turn to natural products with low toxicity and drug resistance. Curcumol (for its structure, see Figure 1A), a guaiane-type sesquiterpenoid hemiketal, is one of the major components of the essential oil of *Rhizoma Curcumae*, which is a common traditional Chinese medicine (TCM). It exhibits characteristics such as anti-hepatic fibrosis [18], anti-inflammatory [19], and importantly, as an anti-proliferative compound triggering a loss in viability in many tumor cell lines with low cytotoxicity [20]. However, little is known about the characteristics and molecular mechanisms of curcumol-triggered cell death.

For anti-cancer treatments, combinatorial strategies could provide synergistic tumor growth inhibition and reduced systemic toxicity in relation to each of the monotherapeutic regimens alone [21–23]. To the best of our knowledge, no studies have previously reported the effect of the combination of celecoxib and curcumol on NSCLC. Thus, the aim of the present study was to investigate the anti-proliferative effect of combinative treatment with celecoxib and curcumol on human lung cancer *in vitro* and the effect on tumor growth and metastasis *in vivo*, to search a potential efficient comprehensive therapeutics for NSCLC. The mechanism of action was also determined in order to improve the treatment of lung cancer.

## RESULTS

### Combined effect of celecoxib and curcumol on growth of NSCLC cells

Before testing the combinative effects of celecoxib and curcumol treatment, we first performed MTT assay to evaluate the cytotoxicity of single drug monotherapy in two NSCLC cell lines, A549 and H1299, and human bronchial epithelial cell line BEAS-2B. As shown in Figure 1B, low dosage of curcumol had no obvious effect on the viability of all three cell lines, which is consistent with previous reports [24]. To assess the combined effect of celecoxib and curcumol on tumor cell proliferation, MTT assay was also performed. Two NSCLC cell lines were treated with the indicated concentrations of celecoxib alone and its combination with 30 μM of curcumol (celecoxib + curcumol) for 24 h. The data indicated that celecoxib alone promoted decreased cell viability dose-dependently, while celecoxib + curcumol exhibited the strongest anti-proliferation ability, which surpassed the sum effect of celecoxib (Figure 1C). At the concentration of 20 μM or lower, no significant difference of the inhibition rate between groups treated with celecoxib + curcumol and celecoxib was found. While at the drug concentration of 40 μM, celecoxib + curcumol showed huge anti-proliferation ability on A549 cells, with the inhibition rate larger than that of celecoxib (*p* < 0.05). As compared with A549 cells, H1299 cells were found to be more sensitive to celecoxib + curcumol treatment, once the proliferation inhibition rate was 25.5 ± 3.2 (%) since 20 μM of celecoxib. However, no synergistic cytotoxicity was observed in BEAS-2B cells. These results suggest that curcumol has an enhanced effect on celecoxib-inhibited proliferation of tumor cells at a subtoxic concentration without increasing cytotoxicity to normal cells. In this study, we used a subtoxic concentration at which celecoxib alone did not induce significant proliferation inhibition. Therefore, drug dosages of celecoxib (30 μM) and curcumol (30 μM) were chosen for combinative therapy in the following experiments.

Although celecoxib is a COX-2 inhibitor, it has been found to exhibit potent pro-apoptotic activity in cancer cells through a mechanism that is independent of its COX-2 inhibitory activity [25, 26]. Therefore, in order to determine whether the synergistic effect of celecoxib and curcumol on NSCLC cell growth occur in a COX-2-dependent route, other COX inhibitors such as nimesulide (COX-2 inhibitor) or indomethacin (COX-1 inhibitor) were also used in combination with curcumol. As shown in Supplementary Figure 1A and 1B, we found that curcumol exerted no synergistic effect on the anti-proliferative action of COX-2-selective inhibitor nimesulide and the NSAID inhibitor indomethacin in A549 cells. In addition, the inhibition on COX-2 protein expression by celecoxib and curcumol combinative treatment in A549 cells was just similar to that by celecoxib monotherapy (Supplementary Figure 1C). Thus, our data provide definitive proof that the enhanced inhibitory effect on tumor cell growth of the combinative treatment is not a result of COX inhibition.

### Combined effect of celecoxib and curcumol on tumor cell apoptosis

**Figure 2:**
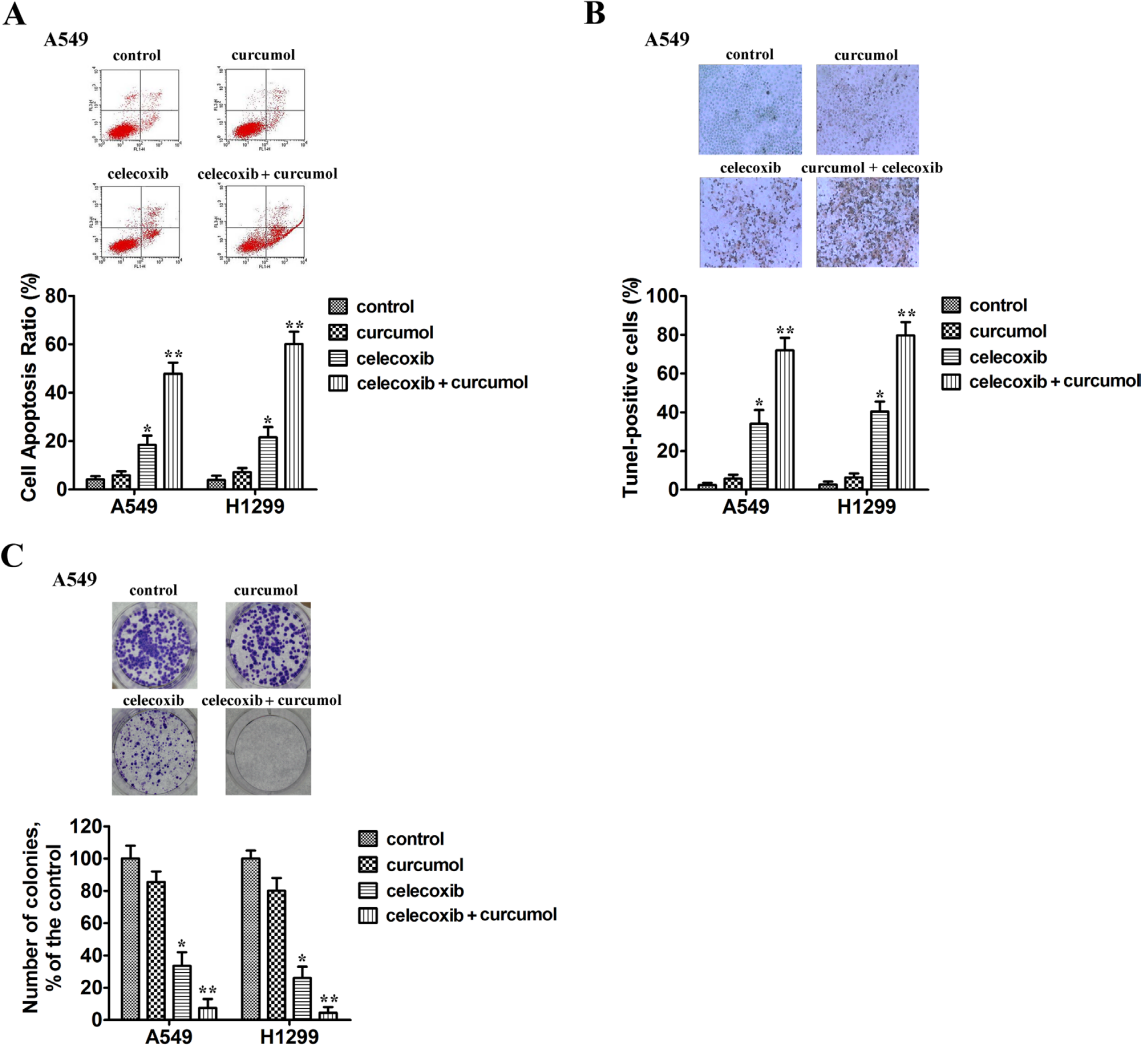
Curcumol enhances celecoxib-induced cell apoptosis and their combination suppresses the clonogenic growth of NSCLC cells **(A)** A549 and H1299 cells were exposed to celecoxib (30 μM) and/or curcumol (30 μM). 18 h later, all cells were harvested for flow cytometry analysis. Annexin V/PI-stained cells were analyzed and the percentage of apoptotic cells was determined. The experiments were carried out independently in triplicate; representative data are shown. Data are represented as mean ± SD. ^*^p < 0.05, ^**^p < 0.01. Annexin V/PI double staining profile of A549 cells is also included. **(B)** A549 and H1299 cells were exposed to celecoxib (30 μM) and/or curcumol (30 μM) for 18 h. TUNEL assays were performed according to the manufacturer’s instructions. The rate of apoptosis was expressed as the percentage of total cells counted. *Each bar* shows the mean ± SD of three independent experiments, performed in triplicate. ^*^p < 0.05, ^**^p < 0.01. TUNEL staining profile of A549 cells is also shown. A *dark brown* DAB signal indicates positive staining, while shades of *blue-green* to *greenish tan* signifies a non-reactive cell. **(C)** Colony formation ability of NSCLC cells treated with celecoxib (30 μM) and/or curcumol (30 μM). The experiments were repeated three times (n = 3); representative data are shown. Data are represented as mean ± SD. ^*^p < 0.05, ^**^p < 0.01. Representative dishes of A549 cells evaluated by colony-forming assay are also included.

To determine whether tumor cellular viability decreased with celecoxib and curcumol *via* apoptosis, we tested the externalization of phosphatidylserine on the cell membrane by Annexin V/PI staining. Two NSCLC cell lines (A549 and H1299) were exposed to celecoxib (30 μM), curcumol (30 μM) or a combination of both. As shown in Figure 2A, after 24 h of treatment, curcumol alone had no obvious effect on tumor cell apoptosis, while monotherapy with celecoxib induced 15-25% apoptosis ratio. However, when A549 and H1299 cells were exposed to combined treatment with celecoxib and curcumol, the number of cells undergoing apoptosis significantly increased (50-65%). This effect was statistically significant as compared to single treatment with either drug alone. TUNEL assays further indicated that curcumol led to an increased apoptotic rate in NSCLC cells treated with celecoxib (Figure 2B). Additionally, colony-forming assay showed that 30 μM celecoxib alone caused mild inhibition of the clonogenic growth of A549 and H1299 cells. In contrast, combined treatment with celecoxib and curcumol markedly suppressed the clonogenic growth of A549 and H1299 cells with inhibition rates of 92.5% and 95.8%, respectively (Figure 2C).

### Celecoxib and curcumol induces apoptosis in a caspase-dependent manner

**Figure 3:**
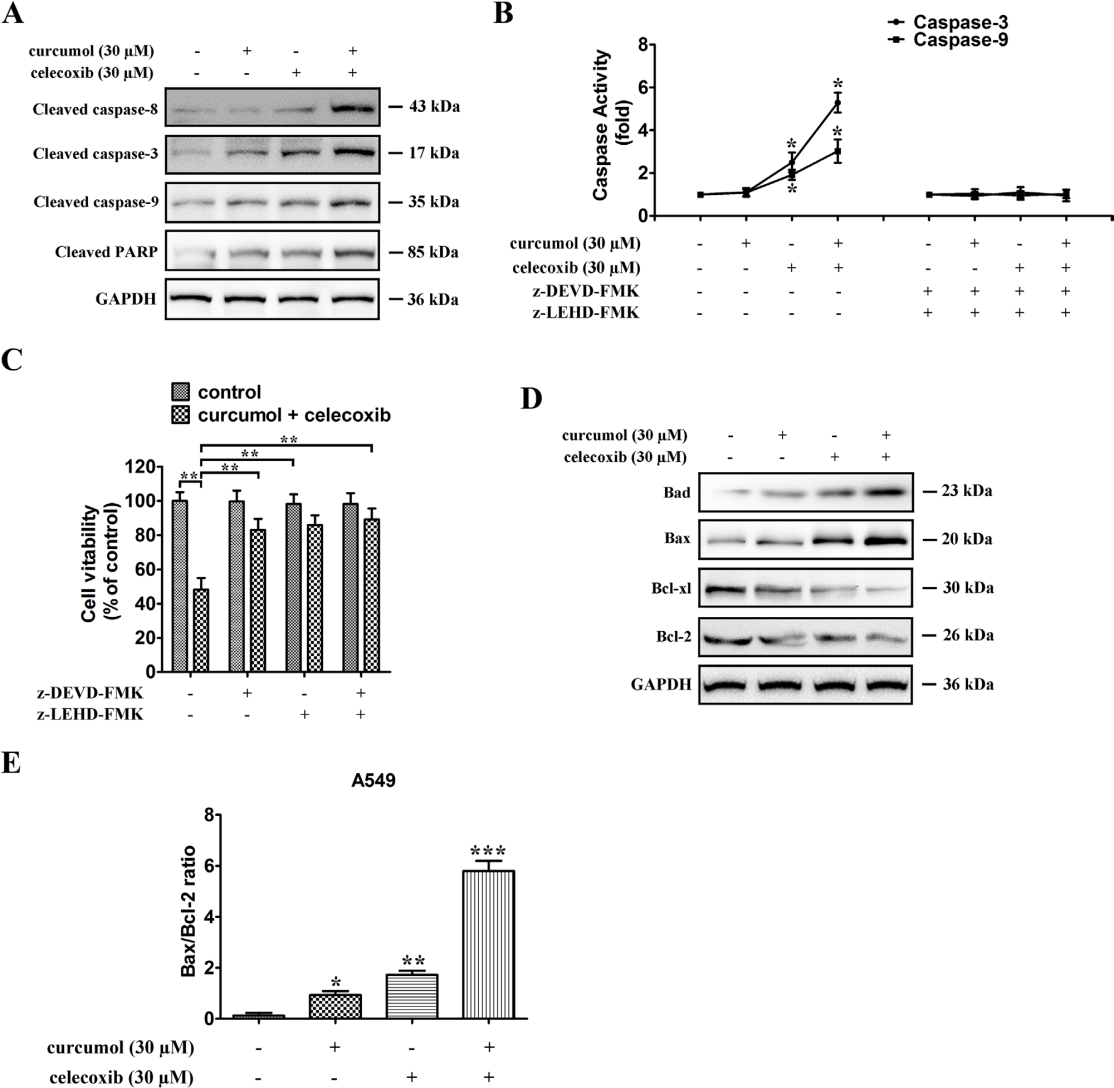
Celecoxib and curcumol-induced apoptosis is mediated through the caspase-dependent mitochondrial pathway in NSCLC cells **(A)** Caspase-8, caspase-9, caspase-3, and PARP expression levels in A549 cells under different treatment conditions. All gels run under the same experimental conditions and the representative images of three different experiments were cropped and shown. **(B)** Activity of caspase-3 and caspase-9 in A549 cells treated with celecoxib and curcumol alone or in combination for 24 h. Data are presented as fold increases as determined by quantitative analysis. ^*^p < 0.05. **(C)** Viability of A549 cells after treatment with caspase inhibitors. Cells were treated with inhibitors for 2 h before the 24 h treatments, after which cell viability was determined by MTT assay. Data are representative of three independent experiments and are represented as mean ± SD. ^**^p < 0.01. **(D)** Expressions of the Bcl-2 family proteins, Bcl-2, Bcl-xl, Bax, Bad, in A549 cells under different treatment conditions. All gels run under the same experimental conditions and the representative images of three different experiments were cropped and shown. **(E)** Band intensity shown in (D) was quantified by Image J software. The ratio of Bax:Bcl-2 was shown. The results shown are representative of three different experiments. Data are represented as mean ± SD, ^*^p < 0.05, ^**^p < 0.01, ^***^p < 0.001.

To explore the mechanisms underlying celecoxib and curcumol-triggered apoptosis in A549 cells, expression of pro-apoptotic proteins and caspases activation were measured by Western blotting assay. Caspases are the main enzymes in apoptotic process. Our results indicated that celecoxib and curcumol by themselves caused minimal proteolytic processing of procaspase-8, -9, and -3. In contrast, celecoxib in combination with curcumol caused an obvious more intensive proteolytic cleavage of procaspase-8, -9, and -3 (Figure 3A). Moreover, celecoxib and curcumol in combination led to the cleavage of PARP, whereas celecoxib or curcumol alone failed to induce PARP cleavage (Figure 3A). Caspase activity, shown in Figure 3B, showed that caspase-3 and caspase-9 activities were respectively 2.5- and 1.9-fold elevated in relation to controls in celecoxib-treated cells and respectively 5.3- and 3.1-fold over that in combinative treatment. Co-treatment with the caspase inhibitors z-LEHD-FMK and z-DEVD-FMK abolished caspase activation triggered by celecoxib and curcumol and rescued A549 cells from treatment-induced apoptosis (Figure 3B). Cell viability was also elevated by caspase inhibitors following combinative treatment (Figure 3C). These observations suggest that activation of a caspase-involved apoptotic pathway might be one of the major mechanisms through which curcumol exerts its synergistic effect on celecoxib-treated A549 cells.

Next, we studied the combinative effect of celecoxib and curcumol on the balance between the anti-apoptotic (Bcl-xl or Bcl-2) and apoptotic (Bad or Bax) members of the Bcl-2 family in A549 cells. The results showed that the combinative treatment also activated the intrinsic apoptotic pathway, as evidenced by an increase in the expression of Bad and Bax and a decrease in the expression of Bcl-xl and Bcl-2 (Figure 3D), resulting in an increase in Bax:Bcl-2 ratio in both cell lines (Figure 3E). The mRNA levels of Bcl-xl and Bcl-2 were also downregulated in A549 cells receiving treatment with the combination of celecoxib and curcumol (Supplementary Figure 2). These results suggest that celecoxib and curcumol combinative treatment induces A549 cell apoptosis through the caspase-dependent mitochondrial pathway.

### Inhibitory effects of celecoxib and curcumol on PI3K/AKT and MAPK activation

**Figure 4:**
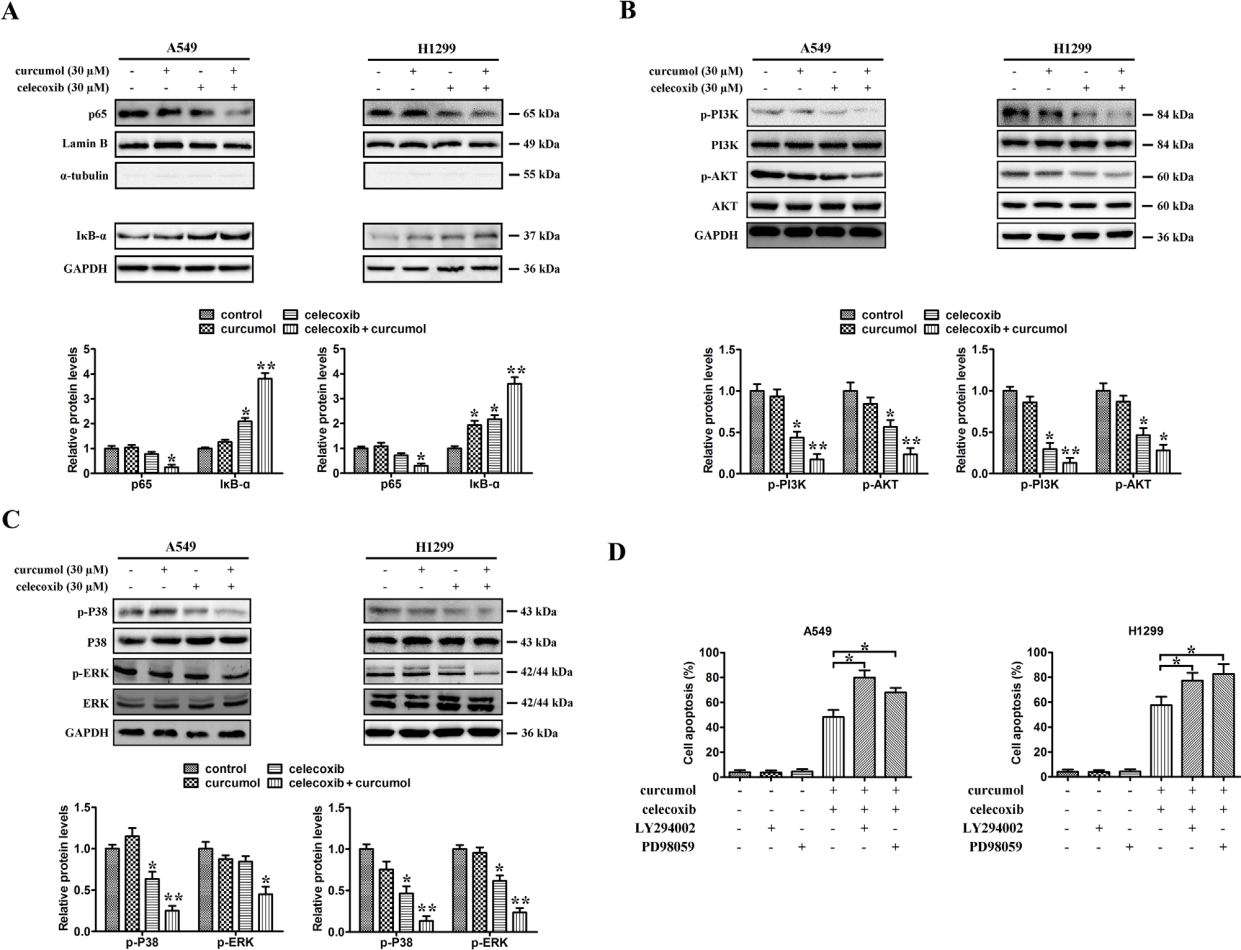
Effects of celecoxib and curcumol on NF-κB, PI3K/AKT and MAPKs signaling pathway **(A)** A549 and H1299 were treated with celecoxib (30 μM) and/or curcumol (30 μM) for 24 h. Nuclear proteins were extracted and subjected to Western blotting for p65 detection. Lamin B was used as loading control. Additionally, the whole cell extracts with the same treatment were prepared and analyzed for IκB-αexpression. **(B)** A549 and H1299 were treated with celecoxib (30 μM) and/or curcumol (30 μM) for 24 h. AKT, p-AKT, PI3K, and p-PI3K proteins in whole cell lysates were determined with specific antibodies. GAPDH was used as loading control. **(C)** A549 and H1299 were treated with celecoxib (30 μM) and/or curcumol (30 μM) for 24 h. Western blotting was performed to detect the levels of p-p38, p38, ERK and p-ERK respectively. Densitometric quantification of the immunoblot data in (A-C) is also shown and data are represented as mean ± SD. ^*^p < 0.05, ^**^p < 0.01. **(D)** The MEK inhibitor PD98059 and PI3K inhibitor LY294002 were used to evaluate whether ERK phosphorylation and AKT inactivation, respectively, are required for apoptosis. The percentages of apoptotic cell death were measured *via* Annexin V/PI staining followed by flow cytometry analysis. Data are represented as mean ± SD, ^*^p < 0.05.

To further investigate the mechanisms behind celecoxib and curcumol-triggered cell death, we assessed changes in the cellular survival pathways in NSCLC cells. Previous studies have demonstrated that NF-κB plays a critical role in cell growth and apoptosis [27, 28]. Therefore, we studied whether celecoxib and curcumol could regulate NF-κB activity in NSCLC cells. The data indicated that low dosage of curcumol alone had no apparent effect on the protein level of NF-κB/p65. However, treatment of celecoxib at the dosage of 30 μM in A549 and H299 cells inhibited the nucleus translocation of p65, which was further enhanced by concurrent exposure to curcumol (Figure 4A). Additionally, 30 μM celecoxib treatment led to increased IκBα levels in A549 and H1299 cells, which was further accentuated upon co-treatment with curcumol (Figure 4A).

Mitogenic and AKT survival pathways have the ability to intensify cellular proliferation, inhibit apoptosis, and potentiate the downstream NF-κB survival pathway [29]. Moreover, several experimental studies have reported that celecoxib-induced apoptosis is associated with the protein kinase AKT [30, 31]. To investigate whether the AKT pathway was involved in celecoxib and curcumol-induced cell death, A549 and H1299 cells were exposed to celecoxib and curcumol alone or in combination for 24 h. As shown in Figure 4B, the phosphorylation levels of AKT and PI3K were markedly decreased after co-treatment with celecoxib and curcumol, but not with curcumol alone. To assess the role of inhibited AKT phosphorylation in the induction of apoptosis following celecoxib and curcumol combinative treatment, the cell-permeable PI3K inhibitor LY294002 was used to inhibit the AKT pathway. As shown in Figure 4D, LY294002 significantly accelerated apoptosis in both two lung cancer cell lines following combinative treatment with celecoxib and curcumol.

Since p38^MAPK^ and ERK also play critical roles in determining cell fate, the effects of celecoxib and curcumol on the activation of these protein kinases were also investigated. As shown in Figure 4C, the phosphorylation levels of ERK were markedly decreased after co-treatment with celecoxib and curcumol, but not with either drug alone. In addition, celecoxib in combination with curcumol reduced phospho-p38 protein level. The specific MAPK/ERK kinase (MEK) inhibitor PD98059 was used in our study to determine whether phospho-ERK1/2 inhibition was required for apoptosis. As shown in Figure 4D, following celecoxib and curcumol combinative treatment, PD98059 significantly accelerated apoptosis in A549 and H1299 cells. Thus, suppression of the survival MAPK ERK in NSCLC cells might also account for the apoptotic effects of celecoxib and curcumol.

### Celecoxib and curcumol combined therapy initiates cell cycle arrest at G0/G1 phase in NSCLC cells

**Figure 5:**
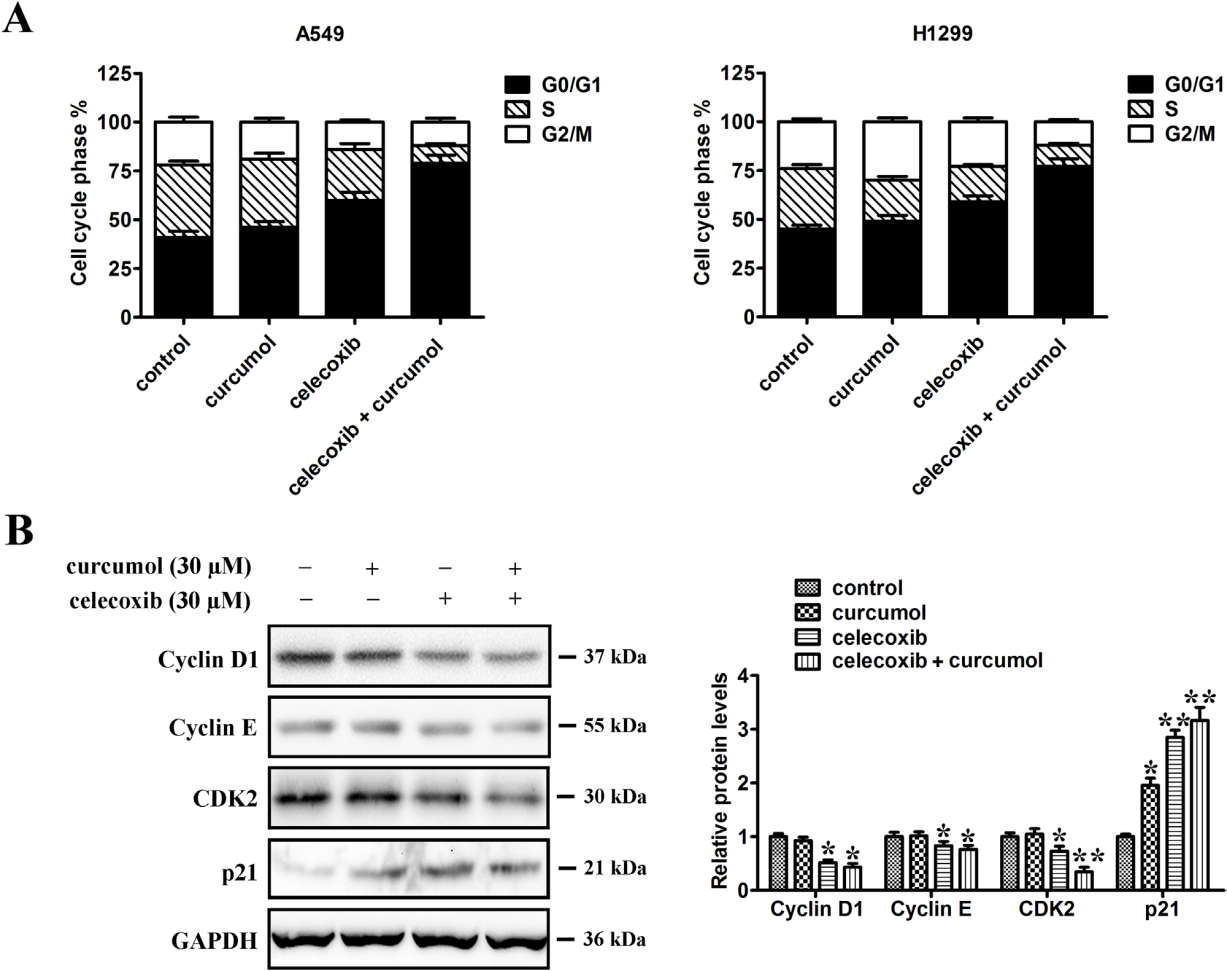
Effect of celecoxib and curcumol on cell cycle distribution **(A)** A549 and H1299 were treated with celecoxib (30 μM) and/or curcumol (30 μM) for 24 h. The cells were then fixed with 70% ethanol and stained with PI. The cell cycle distribution (G0/G1, G2/M and S) was determined by flow cytometry. **(B)** Western blotting was also performed to detect the levels of cell cycle regulators (cyclin D1, cyclin E, cdk2 and p21). Band intensity was quantified by Image J software. The results shown are representative of three different experiments. Data are represented as mean ± SD, ^*^p < 0.05, ^**^p < 0.01.

To examine whether the anti-proliferative effect of celecoxib on lung cancer cell lines was mediated *via* specific cell cycle arrest, we investigated the cell cycle phase distribution by flow cytometric analysis after celecoxib, curcumol or their combined treatment. As shown in Figure 5A, curcumol treatment alone had no obvious effect on cell cycle distribution. However, when A549 cells were incubated with celecoxib, the cell population of G0/G1 phase increased from 41.2% to 60.5% and S phase decreased from 35.3% to 26.2%. When combined with curcumol, we found that there was an obvious accumulation of cells during G0/G1 phase with the ratio of 79.4%, and the cell population of S phase dropped from 26.2% to 9.1% as compared to celecoxib single treatment. Similar effects of celecoxib and curcumol on cell cycle distribution were found in H1299 cells. These results indicate that the major effect of celecoxib on cell cycle is G0/G1 phase arrest, and curcumol can reinforce the cell proliferation inhibition effect of celecoxib by endowing with additional ability of G0/G1 cell cycle arrest.

Cell cycle is mediated by cyclin D1/cdk4 complex at early G1 phase while the G1/S transition is regulated by cyclin E/cdk2 complex. To figure out the potential contributors to the observed G0/G1 cycle arrest, cyclins, p21 and cdk2, which are related to the G1/S checkpoint, were analyzed by western blotting analysis. As shown in Figure 5B, cdk2, cyclin E and cyclin D1 were downregulated in a similar manner with the treatment of celecoxib alone, as well as in combination of celecoxib and curcumol. In contrast, the protein level of p21 was elevated in response to celecoxib monotherapy or the combined treatment, which seems to have negatively regulated those cell cycle checkpoints surpassing proteins.

### Combined effect of celecoxib and curcumol on NSCLC cell migration

**Figure 6:**
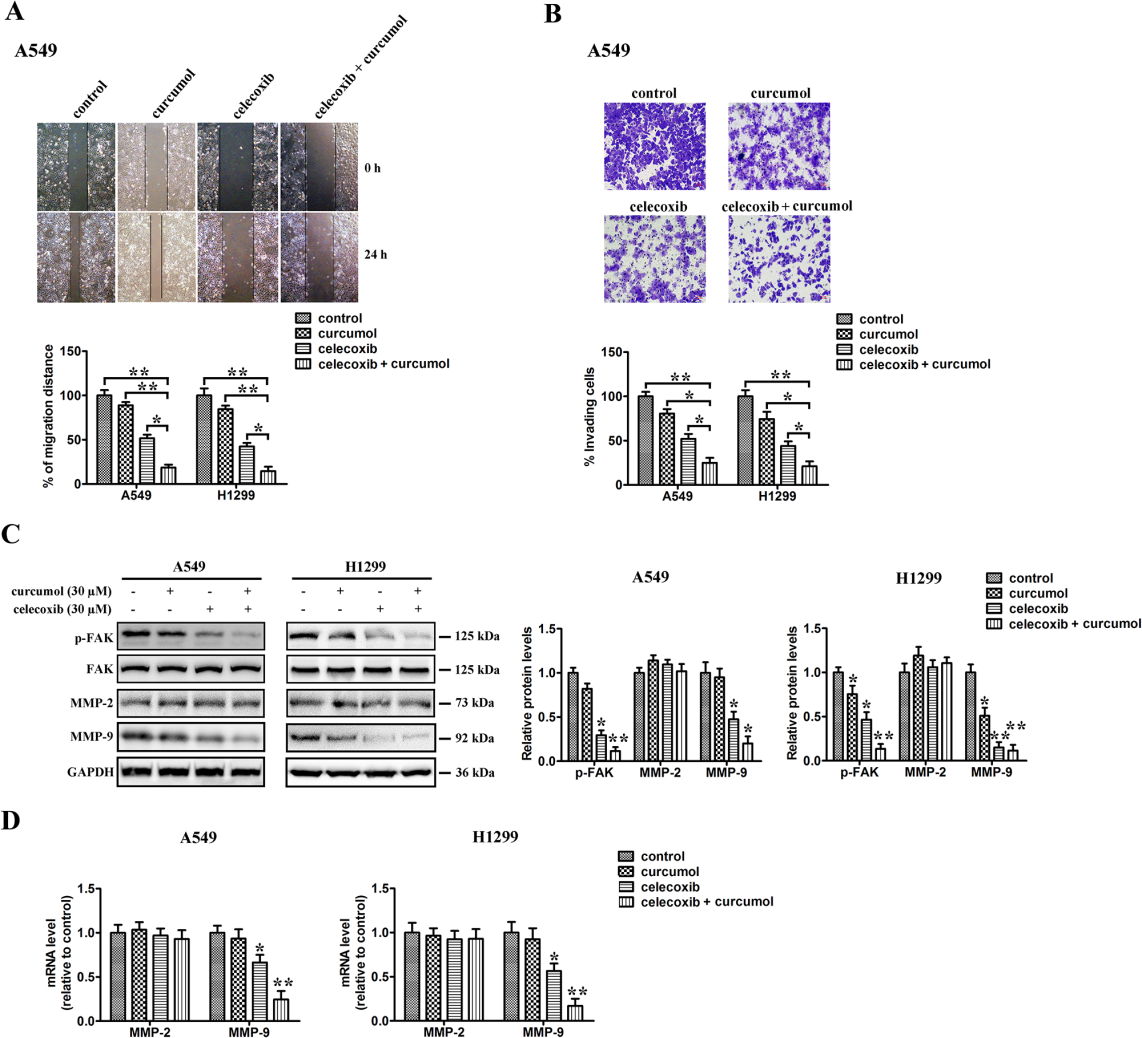
Effect of celecoxib and curcumol on cell migration **(A)** Wound healing assays. A549 and H1299 cells were treated with celecoxib (30 μM) and/or curcumol (30 μM). Photographs were taken immediately and after 24 h of creating the scratch. Images shown are representative of three independent experiments. **(B)** Transwell assay. A549 and H1299 cells were treated with celecoxib (30 μM) and/or curcumol (30 μM). After 16 h pretreatment and 9 h incubation in the upper chamber, the cells migrating to the lower membrane were stained and counted in five fields with a magnification of × 100. N = 3, bar = 50 μm. The experiments were carried out in triplicate and representative data are shown. **(C)** Effects of celecoxib and curcumol on FAK, p-FAK, MMP-2 and MMP-9 protein expression. A549 and H1299 cells were treated with celecoxib (30 μM) and/or curcumol (30 μM). 24 h later, cells were harvested for Western blotting analysis using indicated antibodies. The level of GAPDH served as the loading control. Band intensities were calculated using software Image J. Relative intensities are also shown. Data represent mean values of triplicate samples. **(D)** A549 and H1299 cells were treated with celecoxib (30 μM) and/or curcumol (30 μM). 24 h later, cells were harvested for RNA extraction and quantitative real time PCR using primers specific for human MMP-2, MMP-9, and GAPDH (internal control). Data represent mean values of triplicate samples. ^*^p < 0.05, ^**^p < 0.01.

To investigate the effect of celecoxib and curcumol on tumor cell migration, wound healing and transwell assays were conducted. Wound healing scratch assay showed that, 24 h after the scratch, A549 cells migrated into and largely covered the original wound area, whereas those treated with celecoxib and/or curcumol failed to cover a substantial portion of the wound. Similar results were also observed in H1299 cells (Figure 6A). The transwell motility chamber assay was also performed to examine whether celecoxib and curcumol could inhibit NSCLC cell migration. As shown in Figure 6B, the cells migrating to the lower membrane were stained and quantified. We found that, A549 and H1299 cells treated with celecoxib alone exhibited a mild inhibition in the cell invasiveness, and the combination of celecoxib and curcumol led to enhanced inhibition in NSCLC cells. To explore the mechanisms underlying celecoxib and curcumol combined effect on cell migration, immunoblotting was further accessed to determine the protein level of FAK, the activation of gelatinases, such as MMP2 and MMP9, which have been demonstrated to play important roles in cell migration. The results indicated that combined treatment on NSCLC cells with celecoxib and curcumol markedly decreased phosphorylation level of FAK, while total FAK protein remained unchanged. On the contrary, curcumol alone had no apparent effect on the phosphorylation level of FAK. Similar results were observed in MMP-9 protein expression. Decreased expression level of MMP-9 was found in co-treated cells, compared with celecoxib or curcumol treatment alone (Figure 6C). Consistently, qPCR results indicated that combination of celecoxib and curcumol decreased the mRNA level of MMP-9 in A549 and H1299 cells (Figure 6D). However, no apparent inhibitive effect on protein or mRNA level of MMP-2 was observed in cells receiving combinative treatment with celecoxib and curcumol (Figure 6C and 6D). Collectively, these results indicate that celecoxib and curcumol antagonize the activation of FAK and MMP-9 in NSCLC cells to inhibit cell migration and invasion.

### Combination of celecoxib and curcumol retards the development of lung cancer xenografts in nude mice

**Figure 7:**
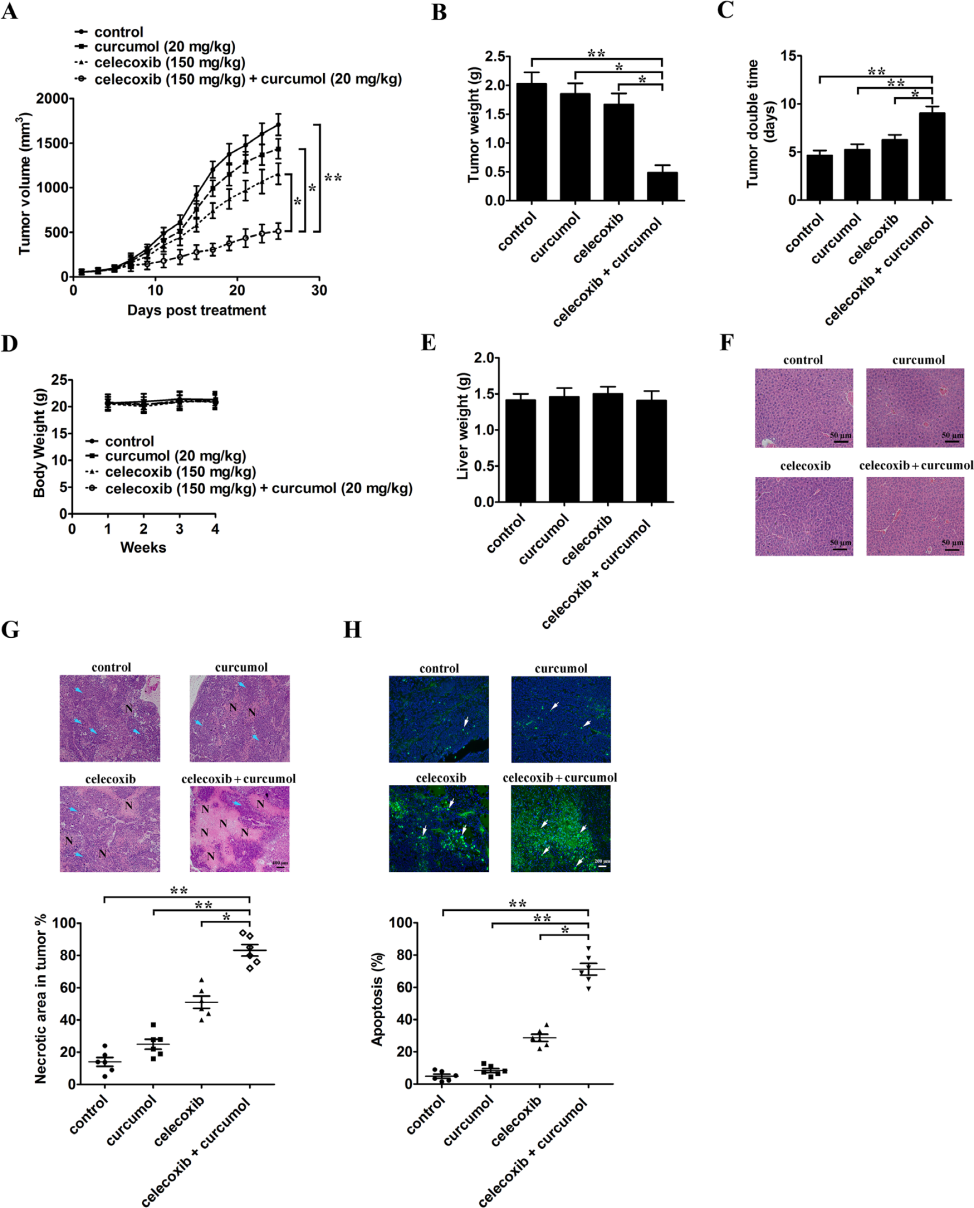
Celecoxib and curcumol combined therapy inhibits *in vivo* tumor xenograft growth in a subcutaneous tumor model A549 cells were injected subcutaneously into the dorsal flanks of athymic nude mice. When tumors reached a size of approximately 50 mm^3^, mice were i.g. with celecoxib and i.p. with curcumol or the combination of two drugs every other day for a total of 25 days. **(A)** The tumor growth inhibitory effects of different treatments were compared. **(B)** At the end of the study, the excised tumors from each group were weighed. **(C)** Tumor double time of each group. **(D)** The weight of nude mice from each group did not change significantly during the experiment. **(E)** Liver weight of mice at the end of the experiment show in (A). **(F)** Representative photomicrographs of liver sections stained with H&E of mice treated with PBS, celecoxib, curcumol, or celecoxib + curcumol. **(G)** Determination of tumor necrosis after combined treatment with celecoxib and curcumol. Tumor necrosis areas are shown by H&E staining and observed under light microscope (×100). The viable tumor cells are indicated by a *blue arrow*. Tumor necrosis was determined by software Image J. Two sections/mouse and three mice were prepared. **(H)** Determination of apoptosis after combined treatment with celecoxib and curcumol. TUNEL assay was used to detect apoptotic cells (stained green and indicated with blank arrows) (× 200). The ratio of apoptotic cells to total cells: TUNEL positive cells were counted from three fields of the highest density of positive-stained cells in each section to determine the percentage of apoptotic cells. All data are shown as mean ± SD. ^*^p < 0.05, ^**^p < 0.01.

The antitumor effect of celecoxib and curcumol was measured in a xenograft tumor model by transplanting A549 cells into athymic nude mice. On the 8^th^ day post-implantation, before the tumor was palpated, mice were randomly divided into 4 groups with at least 8 tumor-bearing mice in each group. Tumor volume was markedly decreased after combinative treatment with celecoxib and curcumol for 25 days as compared to celecoxib or curcumol monotherapy (Figure 7A). Celecoxib alone also inhibited the growth of xenograft tumors to some extent, but the effects were not as significant as those observed in the combinative treatment group. At the end of the study, tumors for each group were removed and weighed. Combinative treatment with celecoxib and curcumol significantly decreased tumor weight compared with the control group, celecoxib or curcumol monotherapy (Figure 7B). Tumor doubling time (TDT) was extended from 4.63 days in mice receiving PBS, 5.23 days in mice receiving curcumol, 6.26 days in mice receiving celecoxib to 9.03 days in mice receiving celecoxib + curcumol (CI = 1.97; Figure 7C), suggesting a synergistic effect of celecoxib and curcumol. Additionally, treatment of mice with celecoxib alone, curcumol alone, or celecoxib and curcumol in combination induced no obvious toxicity and we observed no apparent change in their body weight (Figure 7D), or liver weight (Figure 7E), indicating an absence of hepatomegaly. H&E staining of the liver from mice treated with celecoxib, curcumol or celecoxib + curcumol displayed normal histology as compared with control mice (Figure 7F), suggesting an absence of hepatic toxicity induced by celecoxib and/or curcumol.

H&E staining indicated that the tumor tissues from mice receiving celecoxib and curcumol treatment showed more severe necrosis than celecoxib or curcumol monotherapy (Figure 7G). Tumor necrotic area increased from 14.3% in mice receiving PBS, 25.3% in mice receiving curcumol, 51.8% in mice receiving celecoxib to 83.5% in mice receiving celecoxib + curcumol (Figure 7G). Furthermore, TUNEL assay also indicated that the combinative treatment of celecoxib and curcumol inhibited A549 tumors through induction of apoptosis *in vivo* (Figure 7H).

### Combination of celecoxib and curcumol decreases NSCLC metastasis in mice

**Figure 8:**
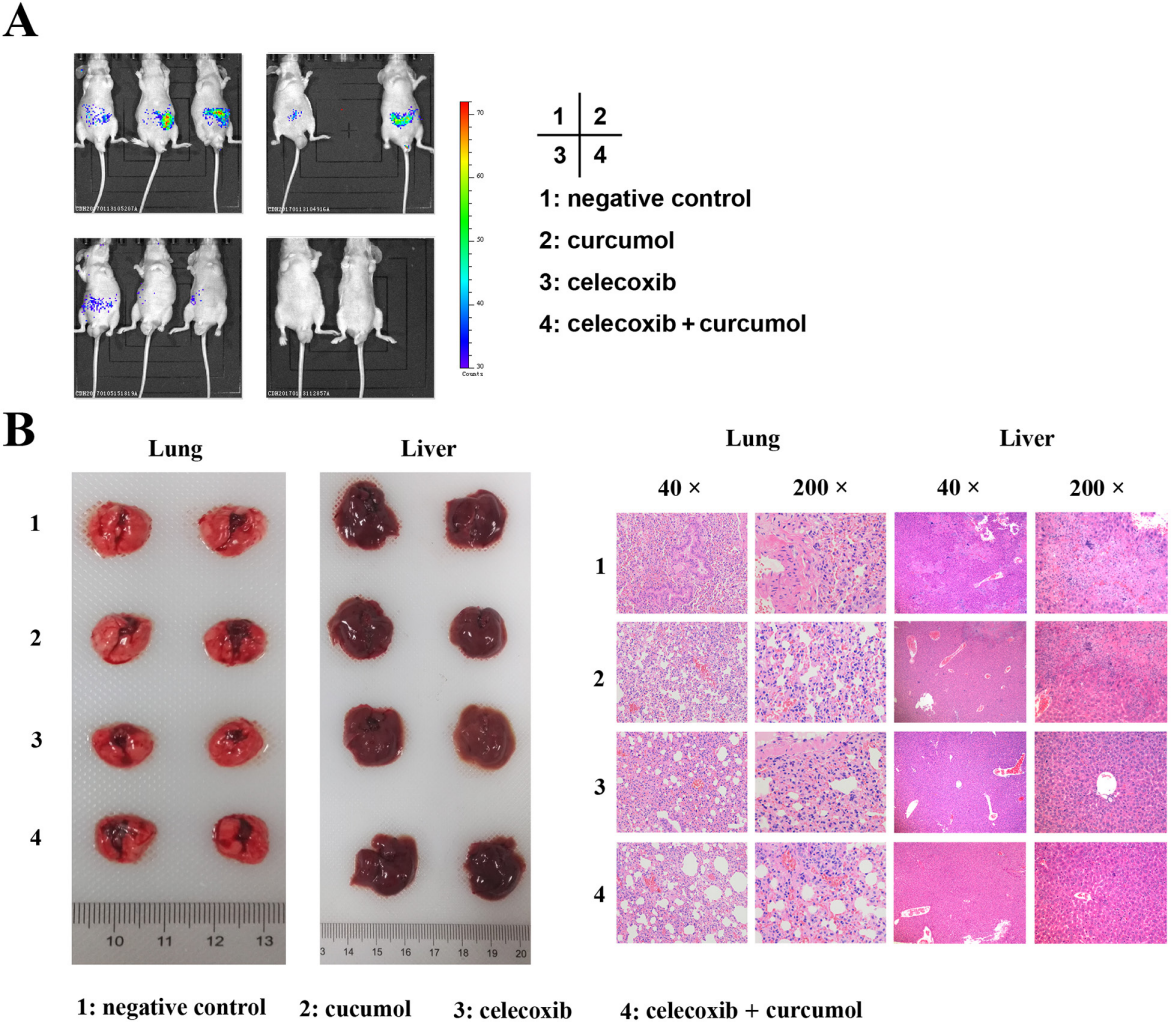
Celecoxib and curcumol combined therapy inhibits tumor metastasis in tail vein injection mouse model A549 cells-xenografted nude mice (n = 8 per group) were i.g. with celecoxib and i.p. with curcumol or the combination of two drugs five times a week for a total of 6 weeks. **(A)** Bioluminescence imaging of whole bodies of nude mice after injection of A549-C8-luc for 35 days using the IVIS system. **(B)** Representative whole organ imaging and H&E tissue staining of nude mice injected with A549 cells. Lung (left) and liver (right) images with corresponding pathological analyses after tail vein injection for 6 weeks.

Since both lung cancer cell migration and invasion are significantly inhibited by celecoxib and curcumol combinative treatment *in vitro*, we therefore examined the effect of celecoxib and curcumol treatment on metastasis *in vivo*. Mice injected with A549 cells through the tail vein were used as a model for NSCLC metastasis. As shown in Figure 8A, the bioluminescence images showed that the mice receiving celecoxib and curcumol combinative treatment had little organs metastases with less bioluminescence than the mice receiving curcumol or the control mice. The histological sections of lungs and livers from mice co-treated with celecoxib and curcumol also displayed smaller metastatic lesions (Figure 8B). These data indicate that the combination of celecoxib and curcumol could be a potential lung cancer therapeutic strategy for targeting lung cancer progression.

## DISCUSSION

It is well established that cancer is a disease manifested by multiple dysregulated signaling pathways, and treatment with a single agent is rarely effective. Combinatorial therapies that target multiple signaling pathways might provide improved therapeutic responses over monotherapies, since multiple survival pathways are activated in transformed cells [32, 33]. Therefore, current therapeutic strategies for cancer are exploring multi-targeting drugs or a combination of drugs to target multiple cell signaling pathways [34].

Incidence and mortality of NSCLC are continuously increasing, making it the leading cause of cancer death all over the world [1, 35]. A number of clinical data indicate that NSCLC has little impressive symptoms until it is well-advanced, which remains poor prognosis [36]. Current therapeutics for NSCLC are chemotherapy and radiotherapy, both of which have their limitations such as low selectivity, toxicity, resistance and serious systemic side-effects [37], and only a small subset of patients survive for more than a year after treatment. Thereafter, novel approaches and new agents are needed to improve the response to traditional treatments in NSCLC patients.

COX-2 inhibitors have been shown to trigger apoptosis of NSCLC cell lines and to enhance the activity of standard chemotherapeutic agents, providing the rationale for combining celecoxib with chemotherapy in NSCLC therapy [38]. Celecoxib is a selective COX-2 inhibitor and has been reported to exhibit anti-tumorigenic effects in multiple types of human cancer cells and animal models [7–9]. Treatment with celecoxib has been shown to act on various pathways and targets in tumor cells, such as apoptosis, proliferation, invasion and angiogenesis through COX-2-dependent and -independent mechanisms [10, 11]. Until now, the pro-apoptotic effects of celecoxib have been proved to be related to various intracellular signaling pathways, such as Akt, NF-κB and caspases, which interact to regulate apoptosis [39]. One limitation for the therapy of cancer by celecoxib is that dosages required to induce apoptosis of cancer cells are high, and clinical use at essential dosages is usually related to serious side effects [40]. Thus, the use of celecoxib as an anti-cancer drug was limited due to concerns associated with toxicity, necessitating the identification of agents with which it could be combined to reduce the amount of drug needed for cancer cell killing efficacy.

A lot of crude medicine has been applied to clinical therapy due to their effectiveness and little side effects. Curcumol, a pure monomer, extracted from the TCM *Rhizoma Curcumae*, has recently been shown to exhibit anti-cancer effects on many cancer cells [20, 41]. Though Zhang’s study [20] reported that curcumol could trigger apoptosis in lung cancer ASTC-a-1 cells *via* caspase-independent pathway, the anti-cancer mechanism is still unclear. In addition, the potential application of curcumol in combination with other anti-cancer drugs has not been thoroughly explored. Since both celecoxib and curcumol exhibit antitumor activity, we formulated the hypothesis that combined therapy with these two drugs increases the effectiveness as compared with single therapy. In the present study, we evaluated the effects of celecoxib and curcumol alone and in combination on cell proliferation, invasion, migration, cell cycle arrest and apoptosis induction in NSCLC cell lines, and investigated the possible underlying mechanisms.

By MTT assay, we observed that curcumol inhibited the proliferation of celecoxib-treated tumor cells synergistically. Compared to each drug alone, low dosage of celecoxib and curcumol in combination triggered substantial apoptosis of cancer cells. It is well-known that cell apoptosis is a kind of programmed cell death regulated by several signaling pathways and finally executed by caspase-3. To further figure out the anti-cancer mechanism of celecoxib and curcumol, we examined the activation of caspase-8, -9 and -3. Our data showed that induced apoptosis of NSCLC cells by celecoxib and curcumol in combination was mediated *via* the activation of caspase-8/caspase-9/caspase-3. Subsequently, caspases activation led to PARP cleavage, nuclear condensation, and finally, the induction of apoptosis. The Bcl-2 family proteins play pro-apoptotic (Bak, Bid, Bax) or anti-apoptotic (Bcl-2, Bcl-xl) roles in the mitochondrial apoptosis pathway by controlling the permeability of the outer mitochondrial membrane. Upregulation of Bax:Bcl-2 ratio will result in the release of some pro-apoptotic proteins from mitochondria [42]. In our study, combinative treatment with celecoxib and curcumol could cause maximum downregulation of Bcl-xl and Bcl-2 with a concomitant upregulation of Bad and Bax so as to cause an elevation in the Bax:Bcl-2 ratio for activating the caspases associated with the mitochondrial cascade for apoptosis in A549 cells. Therefore, curcumol might sensitize A549 cells to celecoxib-induced apoptosis through its effect on the caspase-involved apoptotic pathway and the intrinsic apoptotic pathway.

The nuclear factor-κB (NF-κB) is a pleiotropic transcription factor of many functions involved in cancer cell proliferation, apoptosis and migration [43]. Increased NF-κB activity has been implicated in various types of tumor, such as colon, breast, lung, pancreatic, and bladder cancers [44–46]. Previous study has found that celecoxib could inhibit cell growth, induce apoptosis and alter cell cycle distribution by blocking NF-κB signaling in MDA-MB-231 cells [47]. In this study, we found that celecoxib treatment alone or combinative treatment attenuated the degradation of IκBα and nuclear translocation of p65. Suppression of NF-κB will result in downregulation of the NF-κB-controlled anti-apoptotic proteins, such as c-FLIP, Bcl-xl and Bcl-2, thereby promoting apoptosis [48]. Indeed, combinative treatment with celecoxib and curcumol significantly reduced the expression levels of Bcl-xl and Bcl-2, and eventually resulted in cell apoptosis. Thus, the inhibition of NF-κB by curcumol could provide effective way to sensitize NSCLC cells to celecoxib-triggered apoptotic cell death.

Mitogenic and AKT survival pathways have the ability to intensify cellular proliferation, inhibit apoptosis, and potentiate the downstream NF-κB survival pathway [29]. Activation of AKT promotes cell survival *via* activation of NF-κB. Moreover, several experimental studies have shown that celecoxib-induced apoptosis is related to AKT [30, 31], suggesting that suppression of AKT also contributes to the anti-proliferative activity of celecoxib in certain carcinomas. Recently, Chen *et al*. [49] reported that celecoxib could enhance the sensitivity of lung cancer cells to the EGFR-tyrosine kinase inhibitor ZD1839 by inhibiting AKT signaling. In this study, we found that celecoxib combined with curcumol did not affect the total expression of AKT and PI3K, but significantly decreased the phosphorylation levels of both kinases. Importantly, our study provided strong evidence to support the conclusion that the enhanced apoptosis observed with combinative treatment (celecoxib and curcumol) and a PI3K inhibitor (LY294002) is related to a decrease in phosphorylated AKT. Thus, the suppression of the PI3K/AKT pathway might be an important mechanism underlying the effects of celecoxib combined with curcumol in human NSCLC cells. The inactivation of AKT might result in transcriptional suppression of NF-κB, and the previously well-characterized downregulation of Bcl-xl and Bcl-2 expression by inactivated NF-κB.

MAPKs (mitogen-activated protein kinases) including extracellular signal-regulating kinase (ERK), p38 MAPK and c-Jun N-terminal protein kinase (JNK), play important roles in cell proliferation, apoptosis, and many other events [50]. More and more evidence indicates that alterations of the activities of MAPKs are involved in the effects of antitumor agents in multiple cancer cell lines [51, 52]. Therefore, targeting of the MAPK pathways is a promising strategy for NSCLC therapy. In the current study, we provided evidence that ERK signaling inhibition was an important step in the induction of combined treatment-triggered apoptosis in both NSCLC cell lines. Furthermore, our study provided important evidence that enhanced apoptosis occurred in combination-treated NSCLC cells treated with the MEK1 inhibitor (PD98059), which suggests that combinative treatment with celecoxib and curcumol plays a central apoptotic role in cancer cells, probably by suppressing MAPK signaling pathways.

Cell proliferation is the result of a rapid shift from a quiescent state to the progression of the cell cycle [53]. Flow cytometric results showed that celecoxib alone, or the combination of celecoxib and curcumol induced A549 and H1299 cell cycle arrest at the G0/G1 phase. Control of G1/S transition is regulated by several specific cyclin complexes and cyclin dependent kinases (CDKs) which sequentially activated and degraded to ensure both entry and progress in the cell cycle [54]. For example, cyclin D associates with cdk6 and cdk4 at early G1, whereas cyclin E activates cdk2 during G1/S transition [55]. The p21, a cyclin-dependent kinase inhibitor (CKI), suppresses all cyclin/CDK complexes during the G1 phase and has been reported to be related to cell growth arrest [56]. In addition, previous studies indicate that celecoxib might suppress cell proliferation and carcinogenesis by decreasing CDKs/cyclins activity or inducing of p27 and p21 expression, resulting in cell cycle arrest [25, 57]. In this study, we observed that celecoxib and curcumol could significantly upregulate the protein level of p21 which further resulted in the negative regulation of G1/S transition and subsequently proved to be hazardous for cancer cell growth. Cyclin D1, cyclin E and cdk2 are all factors that could trigger cells through the G1/S transition and their reduction might contribute to the arrest of cells observed in the G1/S checkpoint and induction of DNA damage. In addition, NF-κB might also play a crucial role on cell cycle, as it has been reported in previous studies that cyclin D1 promoter region has binding sites for NF-κB subunits [58], which may positively regulate its mRNA expression. Thereafter, we may reasonably state here that the inhibition of NF-κB could partially contribute to cell cycle arrest induced by combined treatment with celecoxib and curcumol.

Invasion and metastasis are the main causes for poor prognosis, recurrence and death in NSCLC patients. Most patients do not die due to local complications of the primary tumor growth, but rather due to the spread of tumor. Therefore, metastasis is one of hallmarks of malignant tumor and a leading cause of death among cancer patients. Several studies have reported that celecoxib inhibits adhesion and invasion in oral cancer, gastric cancer, colon cancer, lung cancer and osteosarcoma cells through various cell signaling pathways such as NF-kB, MMP-2/9, E-cadherin, β-catenin and AKT [59, 60]. Additionally, inhibition of cell migration is an important goal of a successful anti-cancer therapeutic strategy in lung cancer. In this study, we found that combinative treatment with celecoxib and curcumol dramatically reduced cell migration in both NSCLC cell lines. Further investigations into the molecular mechanism of action indicated that combination therapy decreased the expression of MMP-9 but not MMP-2 in A549 and H1299 cells, which are mainly related to the metastasis process. In contrast, celecoxib or curcumol single therapy had no or slight effect on MMP-9 expression, suggesting that the expression of MMP-9 is regulated by celecoxib and curcumol synergistically. Although MMP-2 shares fairly structure features and broad substrate specificity with MMP-9, both enzymes differ considerably in terms of transcriptional regulation. The 5′ flanking sequence of MMP-2 gene harbors SP-1 binding sites, while the expression of MMP-9 is mainly regulated by NF-κB [61]. The combinative effect of celecoxib and curcumol on MMP-9 expression is probably *via* NF-κB suppression. This might be one of the reasons for different effect of celecoxib and curcumol on the expression of MMP-9, as compared with MMP-2.

To extend the observations made in cultured cells, we determined the combinative treatment of celecoxib and curcumol on the growth of lung cancer xenograft in nude mice. Our results showed that the combinative treatment caused a significant inhibition of tumor growth in xenograft models. Tumors in the single drug-treated groups continued to grow throughout their treatment courses. Meanwhile, no general signs of toxicity or deaths were observed in any groups. As evidenced from our H&E and TUNEL staining results, the most effective inhibition of the tumor growth was due to apoptotic cell death, which was induced by combinative treatment with celecoxib and curcumol. In addition to its anti-migration effects *in vitro*, the combination of celecoxib and curcumol was also effective in reducing tumor formation and metastasis in nude mice injected with NSCLC cells *in vivo*. However, other than causing apoptosis, there are probably additional mechanisms through which combinative therapy can inhibit tumor growth; yet these mechanisms have to be elucidated in future.

**Figure 9:**
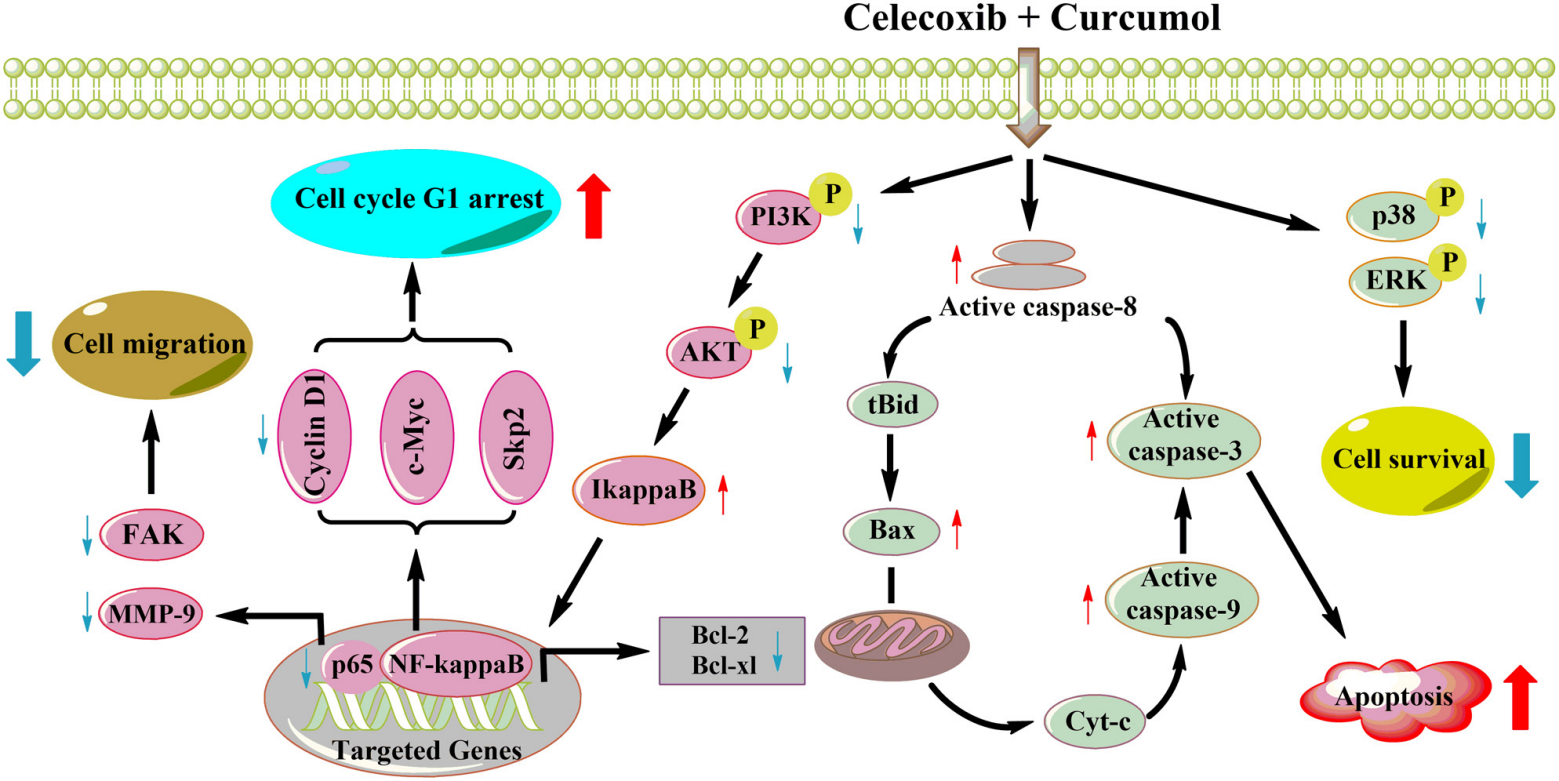
A working model for the synergistic effects of celecoxib and curcumol on NSCLC cells Celecoxib and curcumol cooperatively induce apoptosis through caspase-dependent pathway and cell cycle arrest. In NSCLC cells, combined treatment with celecoxib and curcumol at a low dosage inhibits AKT phosphorylation *via* the PI3K inhibition, which contributes to inhibition of IκBα phosphorylation and degradation, suppresses the nuclear translocation of p65, and, in turn, decreases the expression of NF-κB target genes, such as Bcl-2 and Bcl-xl. Thus, the increase in the Bax:Bcl-2 ratio induces the depolarization of the mitochondrial membrane with the release of cytochrome c and the consequent activation of caspase-9 and caspase-3, resulting in apoptosis of NSCLC cells. Inhibition of NF-κB also results in aberrant expression of cell cycle regulators, such as cyclin D1, cyclin E, cdk2 and p21, which leads to cell cycle G1 arrest and finally promotes cell apoptosis. In addition, the suppression of NF-κB leads to decreased expression of MMP-9 and phospho-FAK, thereby inhibiting tumor cell migration and invasion. Moreover, the combination of celecoxib and curcumol also suppresses the activation of MAPK pathway. All of the above may account for the synergistic effects of celecoxib and curcumol on NSCLC cells.

In conclusion, the present study provides convincing evidence that the combinative treatment with celecoxib and curcumol significantly reduced NSCLC cell proliferation, migration and invasion and induced cell apoptosis *in vitro* and inhibited A549 tumor growth and metastasis *in vivo*, whereas the single treatments did not markedly improve the anti-cancer effect. Further investigations into the molecular mechanism of action showed that celecoxib in combination with curcumol enhances the anti-proliferative and pro-apoptotic effects on NSCLC cells *via* actions on the anti-apoptotic AKT and ERK signaling pathways (Figure 9). Curcumol strengthens the anti-proliferative action of celecoxib, promoting NSCLC cell apoptosis and allowing for the use of lower dosages of celecoxib than those currently used. Therefore, it is worthwhile to consider this combinative treatment for NSCLC and warrants further evaluation in clinical trials.

## MATERIALS AND METHODS

### Cells, cell culture, and reagents

NSCLC cell lines A549 and H1299, and human bronchial epithelial cells (BEAS-2B cells) were purchased from the American Type Culture Collection (ATCC, Philadelphia, PA, USA). Human A549 lung adenocarcinoma cell line stably transfected with luciferase (A549-C8-luc) was purchased from Caliper Life Sciences (Alameda, California). NSCLC cells were grown in RPMI 1640 (Invitrogen, Carlsbad, CA, USA) supplemented with 10% (v/v) fetal bovine serum (FBS; Invitrogen, Carlsbad, CA, USA) and 1% penicillin-streptomycin (Invitrogen, Carlsbad, CA, USA). BEAS-2B cells were grown in DMEM (Invitrogen, Carlsbad, CA, USA) supplemented with 10% FBS, 100 U/mL penicillin and 100 U/mL streptomycin (Invitrogen, Carlsbad, CA, USA). All cells were cultured in a humidified CO_2_ incubator at 37°C. Curcumol (purity > 95%) was purchased from Guangzhou Institutes of Biomedicine and Health, Chinese Academy of Science (Guangzhou, China). Celecoxib, indomethacin and nimesulide were kindly supported by School of Pharmaceutical Science, Southern Medical University (Guangzhou, China). PD98059 and LY294002 were purchased from Sigma (St. Louis, USA).

### Cell proliferation assay

The effects of celecoxib, curcumol or the combination of two drugs on cell proliferation were assessed by the MTT assay. Cells in the exponential growth phase were seeded into a 96-well plate at a density of 5000 cells per well. After 24 h, celecoxib (0-160 μM), curcumol (0-60 μM), or the combination of both drugs were added to the medium. The cells were incubated at 37°C for 24h, then the cell viability was determined by the colorimetric MTT [3-(4, 5-dimethylthiazol-2-yl)-2, 5-diphenyl-2H-tetrazolium bromide] assay at 570 nm by TECAN Safire Fluorescence Absorbance and Luminescence Reader (Vienna, VA, USA). The cell viability was calculated according to the formula: Cell viability (%) = average A_570 nm_ of treated group/average A_570 nm_ of control group × 100%. Each experiment was performed in quadruplicate and repeated at least three times.

### Flow cytometry

To quantify the percentage of cells undergoing apoptosis, we used the Annexin V-FITC kit as described by the manufacturer (BD Biosciences, CA, USA). Briefly, A549 and H1299 cells were incubated for 18 h with celecoxib (30 μM) and curcumol (30 μM) alone or with the combination of both drugs. Next, the treated cells were collected and trypsinized for 3-5 min. The digested cells were washed twice with cold phosphate buffered saline (PBS) and resuspended in binding buffer at a concentration of 1 × 10^6^ cells/mL. After incubation, 100 μL of the solution was transferred to a 5 mL culture tube, and 5 μL of Annexin V-FITC (20 μg/mL) and 5 μL of PI (20 μg/mL) were added. The tube was gently centrifuged at 1000 rpm for 5 min and incubated for 15 min at room temperature in the dark. At the end of incubation, 400 μL of binding buffer was added, and the cells were analyzed immediately by flow cytometry (BD Biosciences, CA, USA). Flow cytometry analysis was performed with untreated A549 and H1299 cells as control.

### Inhibition assay

A549 and H1299 cells were cultured with a MEK inhibitor (20 μM PD98059) or PI3K (10 μM LY294002) for 2 h in the dark. Next, celecoxib and curcumol were added for 18 h. The numbers of apoptotic cells were measured *via* Annexin V-FITC/PI staining.

### Colony-forming assay

Colony-forming assay was performed as previously described [62]. Briefly, About 300 cells in log phase were plated into 60 mm tissue culture Petri-dish (Greiner) in triplicate with 3 mL of culture medium and grown at 37°C with 5% CO_2_. After 48 h culture for cell adherence to the plate, rinsed with fresh medium, celecoxib (30 μM), curcumol (30 μM), or celecoxib + curcumol were added to the medium. 48 h later, the cells were washed twice with PBS and then incubated in drug-free medium. The medium was changed every 5 days. After culturing for additional 10-14 days, the medium was discarded and each dish was washed twice with PBS carefully. The cells were fixed with methanol for 15 min and stained with a 1:10 dilution of Giemsa regent (Merck Biosciences, Darmstadt, Germany) for 10 min. Any grouping of cells containing 30 or more cells was counted as a colony using an inverted microscope (Zeiss, 40-fold magnification). Colony numbers were determined from triplicate plates. Colony growth was related to the control value without any treatment.

### TUNEL assays

Exponentially growing cells were treated with celecoxib (30 μM), curcumol (30 μM) or celecoxib + curcumol for 18 h. The TdT-mediated dUTP nick end labeling (TUNEL) assay was performed as previously described [62].

### Caspase activity assay

Caspase-3 and caspase-9 activities were measured as previously described [63].

### Cell cycle analysis

NSCLC cells (2 × 10^6^) were treated with celecoxib (30 μM) and curcumol (30 μM) alone or in combination for 24 h. Then cell cycle was analyzed on a FACSCalibur flow cytometer with CellQuest software (BD Biosciences, CA, USA) as previously described [63].

### Wound-healing assay

Cells were plated in 12-well culture plates to form cell monolayer (near 70% confluence). After serum starvation for 12 h, a wound was made with a sterile P-200 micropipette to scrape off the cells. The wells were then washed three times with PBS to remove non-adherent cells and incubated in the medium containing 10% FBS with celecoxib (30 μM), curcumol (30 μM), or the combination of both drugs for 24 h. The progress of wound closure was monitored with microphotographs of × 10 magnification taken with light microscope (Carl Zeiss Axioplan 2) at the beginning and the end of the experiments after washing with PBS.

### Cell migration assay

The effects of celecoxib, curcumol or the combination on tumor cell migration were also assessed by the transwell assay. The cell migration assay was performed using transwell inserts (8.0 mm pore size, Millipore, Billerica, MA, USA) as described previously [63]. Before the experiment, A549 and H1299 cells had been cultured in serum-free medium with celecoxib (30 μM), curcumol (30 μM), or the combination (PBS used as buffer control) for 18 h. Then the cells were harvested and resuspended in the same medium. 1 × 10^5^ cells in a volume of 0.1 mL were added to the upper chamber, and the lower chamber was filled with 0.6 mL of 20% FBS supplemented medium. After incubation at 37°C for 9h, cells on the upper surface of the membrane were removed. The migrant cells attached to the lower surface were fixed in 10% formalin at room temperature for 30 min, and stained for 20 min with a solution containing 1% crystal violet and 2% ethanol in 100 mM borate buffer (pH 9.0). The number of cells migrating to the lower surface of the membrane was counted in five fields under a microscope with a magnification of × 100. All groups of experiments were conducted in triplicate, and the cell number was counted by Image-Pro Plus 6.0 software.

### Western blot analysis

Whole cell lysate was prepared with RIPA buffer (Santa Cruz Biotechnology) containing protease inhibitors, PMSF and orthovanadate. Supernatants were collected and protein concentration was determined by the Bio-Rad protein assay method (Bio-Rad, Hercules, CA). In addition, nuclear extracts were prepared as described by Schreiber *et al* [64]. Western blotting used standard protocols. Proteins were separated by SDS-PAGE and transferred onto nitrocellulose membranes that were blocked with 5% non-fat milk in TBS containing 0.1% Tween-20, and incubated with primary antibodies: cleaved caspase-8, cleaved caspase-3, cleaved caspase-9, PI3K, p-PI3K p85 (Tyr458), AKT, p-AKT (Ser 473), FAK, p-FAK (Tyr397), MMP-2, MMP-9 (Cell Signaling Technology, Beverly, MA, USA), IкB-α, NF-κB p65 (Invitrogen, Carlsbad, CA), GAPDH, Lamin B, α-tubulin, ERK, p-ERK, p38, p-p38, cleaved poly (ADP-ribose) polymerase (PARP), Bad, Bax, Bcl-xl, Bcl-2 (Santa Cruz Biotechnology, Santa Cruz, CA). Secondary antibodies were coupled to horseradish peroxidase, and were goat anti-rabbit or goat anti-mouse. Bound antibodies were then visualized with ECL plus Western blotting detection reagents (GE Healthcare). Signal intensity was quantified by densitometry using the software Image J (NIH, Bethesda, MD). All experiments were done in triplicate and performed at least three times independently.

### RNA extraction and quantitative real-time PCR

Cells were incubated with celecoxib (30 μM), curcumol (30 μM), or the combination of two drugs for 24h. Total RNA was then extracted from treated cells using a TRIzol reagent (Invitrogen, Carlsbad, CA, USA) following the manufacturer’s instructions and was used to prepare cDNA by PrimeScript RT reagent Kit (Takara, Otsu, Shiga, Japan). Quantitative real-time PCR was performed with SsoFast EvaGreen Supermix on a CFX96 Real-Time System (Bio-Rad Laboratories, Hercules, CA, USA). The sequences of PCR primers used in our study were synthesized commercially, and are shown in Supplementary Table 1. The glyceraldehydes 3-phosphatase dehydrogenase (GAPDH) gene was used as the reference gene. All data were means of fold change of triplicate analysis and normalized with those of GAPDH.

### Animals

Athymic nude mice (6-8 weeks of age) were obtained from Shanghai Laboratory Animal Center (Shanghai, China) and housed under germfree conditions. All animals received human care according to Chinese legal requirements. The experiments were approved by Nanjing University Animal Care and Use Committee (20160122), and we strictly followed these rules during our experiments.

### *In vivo* xenograft tumor model of human NSCLC

A549 cells (5×10^5^ cells in 30 μL) were injected subcutaneously into the dorsal flanks of mice. Tumor volume was monitored by measuring the two maximum perpendicular tumor diameters with callipers every other day. All tumor-bearing mice were divided randomly into 4 groups, and treatment was initiated on the 8^th^ day when the volume of tumor reached a size of approximately 50 mm^3^. The mice were injected intraperitoneally (i.p.) with curcumol (20 mg/kg), or received (i.g.) celecoxib (150 mg/kg), or the combination of two drugs every other day for a total of 25 days. Control mice received i.p. injection of PBS. Antitumor activity of treatments was evaluated by tumor growth inhibition. The formula, tumor volume = length × width^2^ × 0.52 was used to mimic the tumor volume. At the end of study, the tumors were collected and weighed. The body weight and liver mass were also examined to evaluate the toxicity of different treatments *in vivo*.

In a parallel animal assay (totally 4 groups, and 3 mice per group), the tumor establishment and drug treatment are the same as described above. On the 25^th^ day, mice were euthanized. Tumors and livers were collected, fixed with 4% formaldehyde, embedded in paraffin and sectioned for haematoxylin and eosin (H&E) staining according to standard histological procedures. Apoptotic cells in tumor sections (two sections per mouse, three mice in total) were visualized by the TUNEL technique according to the manufacturer’s instruction (Vazyme, Nanjing, China).

### Calculation of tumor doubling time and combination index

The tumor doubling time (TDT) and combination index (CI) were calculated using GraphPad Prism v 5.0. TDT values were generated from exponential growth curves, which had been fitted to % change in tumor volume data (*r*^2^ > 0.70). Our CI calculations were adapted [65] to apply to TDT values. First, the TDT value for untreated mice was subtracted from the TDT value for each treatment group to obtain ‘blanked’ TDT values (TDT_B_). Then, the CI was calculated as the ratio of TDT_B_ values of combination treatment to individual treatments: CI = (TDT_B_ combination of celecoxib and curcumol)/(TDT_B_ celecoxib alone + TDT_B_ curcumol alone).

### Development of tail vein injection lung cancer model

A549-C8-luc cells (2×10^6^) suspended in 200 μL of PBS were injected into the tail vein of the nude mice. Beginning on the second day, the mice were administered 150 mg/kg celecoxib (i.g.), or 20 mg/kg curcumol (i.p.), or the combination of two drugs five times a week for a total of 6 weeks with at least 8 mice per group. The mice were then repeatedly imaged for metastatic tumor spreading to distant organs. Bioluminescent imaging was detected from luciferase-expressing A549 cells (A549-C8-luc) after injection into the mice. Luciferin (Firefly Luciferin, Caliper Lifesciences Corp., USA) was used as the substrate for the luciferase-expressing tumor cells and injected intraperitoneally at a concentration of 150 mg/kg in PBS, 15 min before imaging. Mice were then anesthetized using 2% isoflurane and imaged using a cooled CCD camera (IVIS system, Caliper Lifesciences Corp.). Exposure times ranged from 1 min to 1 s. Images were quantified as photons per second using the Living Image 4.2 software (Caliper Lifesciences Corp). At the end of study, mice were euthanized and the lungs and livers were harvested, fixed in 10% formalin, and paraffin embedded for pathological examination of H&E slides.

### Statistical analysis

Statistical analysis was carried out using the SPSS software (version 11.0; SPSS, Chicago, IL). Data were expressed as the mean ± standard deviations (SD). For paired data, statistical analyses were performed using two-tailed Student’s t-tests. For multiple comparisons, statistical analyses were performed using one-way analysis of variance (ANOVA) with a Tukey post-test. For all analyses, *P* < 0.05 was considered statistically significant.

## SUPPLEMENTARY MATERIALS FIGURES AND TABLES



## Abbreviations

NSCLC: non-small cell lung cancer
NSAIDs: non-steroidal anti-inflammatory drugs
TCM: traditional Chinese medicine
PBS: phosphate buffered saline
FBS: fetal bovine serum
TUNEL: TdT-mediated dUTP nick end labeling
H&E: haematoxylin and eosin
NF-κB: nuclear factor-κB
IκB: inhibitor of κB
MAPKs: mitogen-activated protein kinases
ERK: extracellular signal-regulating kinase
JNK: c-Jun N-terminal protein kinase
CDKs: cyclin dependent kinases
CKI: cyclin-dependent kinase inhibitor
MMPs: matrix metalloproteinases
TDT: tumor doubling time
CI: combination index.
