# Supplementary material for: Curcumol potentiates celecoxib-induced growth inhibition and apoptosis in human non-small cell lung cancer
Source: Oncotarget. 2017 Dec 14;8(70):115526–45. doi: 10.18632/oncotarget.23308 (PMC5777791; doi:10.18632/oncotarget.23308)
Supplement: Supplementary file 1 [file oncotarget-08-115526-s001.pdf]

Curcumol potentiates celecoxib-induced growth inhibition and apoptosis in human non-small cell lung cancer

SUPPLEMENTARY MATERIALS

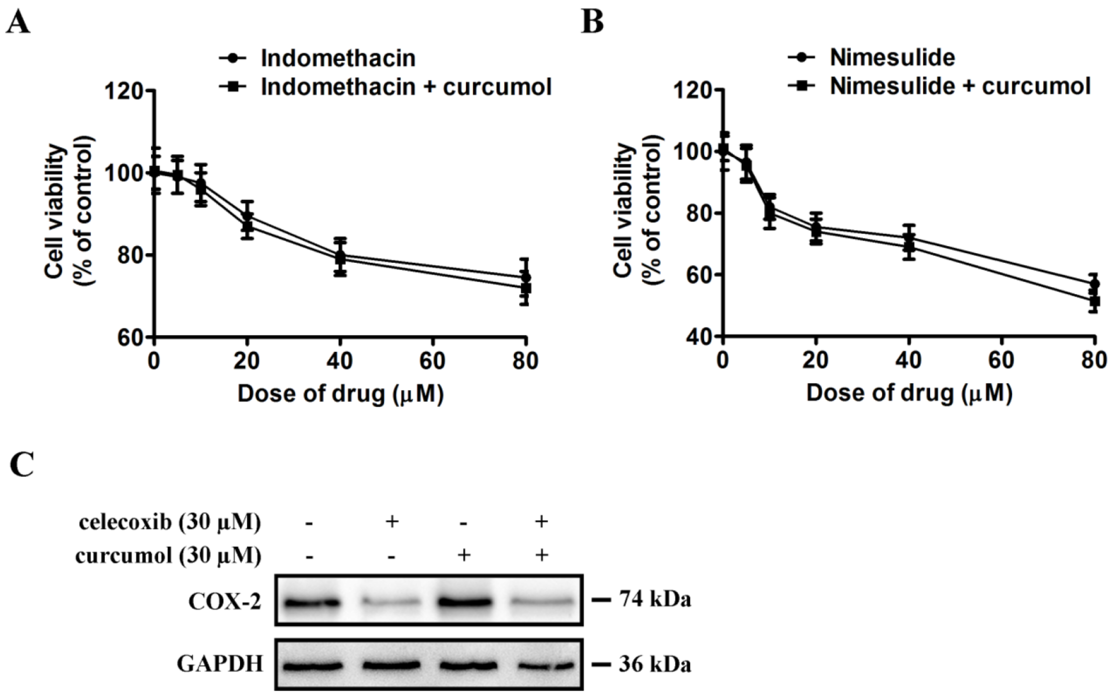

Supplementary Figure 1: Improvement of curcumol on the cytotoxic capacity of celecoxib in NSCLC cells is not associated with COX inhibitory activity. (A-B) Effects of curcumol on indomethacin or nimesulide-induced cytotoxicity in A549 cells. A549 cells were treated for 48 h with indomethacin (A) or nimesulide (B) (0-80 μM) alone or in combination with curcumol (30 μM). Cell viability was then determined by MTT assay. Data are represented as mean ± SD. **(C)** COX-2 expression in A549 cells treated with curcumol (30 μM), celecoxib (30 μM), or their combination for 24 h by western blotting analysis. These results are representative of three independent experiments.

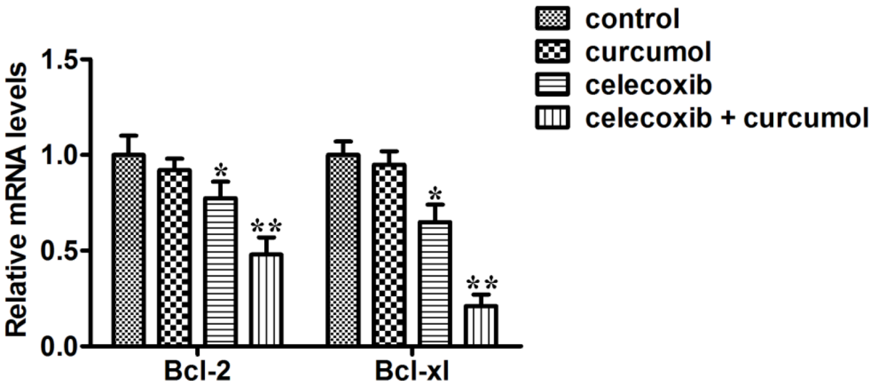

Supplementary Figure 2: Effect of celecoxib and curcumol on Bcl-2 and Bcl-xl mRNA levels. A549 cells were treated with celecoxib (30 μM) and/or curcumol (30 μM) for 24 h. Q-PCR analysis was performed to detect the level of the mRNA transcripts of Bcl-2 and Bcl-xl. The results shown are representative of three independent experiments. The histogram shows the mean ± SD. $^{*}p < 0.05$, $^{**}p < 0.01$.

Supplementary Table 1: The primers of selected genes for real time PCR

Gene	Forward primer (5’-3’)	Reverse primer (5’-3’)
MMP-2	CCGTCGCCCATCATCAA	GGTATTGCACTGCCAACTCTTTG
MMP-9	GGACGATGCCTGCAAGT	ACAAATACAGCTGGTTCCCAATC
Bcl-2	AACATCGCCCTGTGGATGAC	GGCCGTACAGTTCCACAAAG
Bcl-xl	GGCCACTTACCTGAATGACC	AAGAGTGAGCCCAGCAGAAC
GAPDH	AACGACCCCTTCATTGAC	TCCACGACATACTCAGCAC
